# Association of vitamin B1 with cardiovascular diseases, all-cause and cardiovascular mortality in US adults

**DOI:** 10.3389/fnut.2023.1175961

**Published:** 2023-08-31

**Authors:** He Wen, Xiaona Niu, Ran Zhao, Qiuhe Wang, Nan Sun, Le Ma, Yan Li, Wei Zhang

**Affiliations:** ^1^The First Affiliated Hospital, Xi’an Jiaotong University Health Science Center, Xi’an, China; ^2^Department of Cardiology, Tangdu Hospital, Second Affiliated Hospital of Air Force Military Medical University, Xi’an, China; ^3^School of Public Health, Xi’an Jiaotong University Health Science Center, Xi’an, China; ^4^Key Laboratory of Environment and Genes Related to Diseases (Xi’an Jiaotong University), Ministry of Education of China, Xi’an, China

**Keywords:** dietary vitamin B1, cardiovascular diseases, all-cause mortality, cardiovascular mortality, NHANES

## Abstract

**Background:**

The correlation between dietary vitamin B1 intake and cardiovascular diseases, as well as the all-cause and cardiovascular-associated mortality, is not well known. A large-scale data pool was used to examine the aforementioned correlations of Vitamin B1.

**Methods:**

This paper analyzed the dietary data from the survey conducted by National Health and Nutrition Examination (NHANES; 1999–2018). The correlation of vitamin B1 intake in each quartile with cardiovascular diseases such as hypertension, coronary heart disease, myocardial infarction and heart failure was analyzed using multivariate logistic regression models. The hazard ratios for dietary vitamin B1 intake in each quartile, along with all-cause and cardiovascular-associated mortality, were performed using multivariate cox regression analysis, setting the lowest quartile (Q1) as a reference. The restricted cubic spline (RCS) method was used to study the nonlinear relationship. Subgroup stratification and sensitivity analyses were used to further investigate the association between them.

**Results:**

The study enrolled 27,958 subjects (with a mean follow-up time of 9.11 years). After multivariate adjustment, dietary vitamin B1 intake was significantly associated with hypertension, heart failure and cardiovascular mortality, with the most significant association in quartile 4 (Q4) of vitamin B1 intake. The results of the restricted cubic spline showed that vitamin B1 intake was nonlinearly associated with hypertension, whereas it was linearly associated with heart failure and cardiovascular mortality. Meanwhile, a dose–response correlation was observed, indicating that increased vitamin B1 intake leads to reduced risk of both cardiovascular prevalence and mortality. The stratified analysis showed that the correlation between age ≥ 50 years, overweight, smoking history, drinking history and dyslipidemia were more significant in male patients. The associations remained similar in the sensitivity analyses.

**Conclusion:**

The large NHANES-based studies indicate a gradual trend toward decreasing the risk of hypertension and heart failure prevalence and cardiovascular mortality with increasing dietary vitamin B1 intake. This association is especially significant in elderly-aged men, overweight individuals, smokers, drinkers, and dyslipidemia patients.

## 1. Introduction

Globally, cardiovascular diseases (CVD) are one of the foremost causes of death, with a mortality rate of 17.9 million deaths per year (32% of global deaths), and are recognized by the United Nations as a major global health burden (1, 2). Cardiovascular diseases include heart failure, hypertension, ischemic heart disease (stable angina and acute coronary syndrome), cerebrovascular diseases (stroke), valvular abnormalities (aortic stenosis), and arrhythmias (atrial fibrillation), and other various diseases (3–5). Recently, the American Society of Preventive Cardiology (ASPC) published *10 Things to Know About the 10 Major Risk Factors for cardiovascular diseases (2022)*. These 10 factors include unhealthy diet, lack of exercise, dyslipidemia, pre-diabetes/diabetes, hypertension, obesity, special population, thrombosis, renal insufficiency, family history/genetic factors/familial hypercholesterolemia (6). The American College of Cardiology (ACC) and the American Heart Association (AHA) formulated an extensive report on the prevention of cardiovascular diseases (7). The European Society of Cardiology (ESC) also issued strategies to prevent CVD incidence in clinical practice in 2016 (8). The aforementioned report mentioned that healthy nutrition is the cornerstone of cardiovascular disease prevention, and is often one of the most challenging factors in managing cardiovascular disease risk factors. Despite these challenges, nutritionists have determined that even small, but targeted health changes in dietary intake have the potential to improve cardiovascular health (9).

Vitamin B1, also known as thiamine, is an essential micronutrient (10). However, vitamin B1 itself is only stored in small amounts in the body and cannot be produced endogenously, and has a half-life of 1–3 weeks in the body (11). Its main sources are dietary (whole grains, legumes, nuts) or ingested from supplements. In recent years, with the improvement in living conditions, the consumption of white rice has increased, which has increased the incidence of vitamin B1 deficiency. In addition, vitamin B1 is an essential coenzyme required for glucose metabolism. The consumption of more high-calorie diets has increased nowadays which in turn increases the demand for vitamin B1 in the human body, resulting in vitamin B1 deficiency (12). In addition, Excessive alcohol consumption can affect the cellular transport of vitamin B1 and its intracellular phosphorylation (13). Chronic alcoholism has been found to be one of the main causes of vitamin B1 deficiency in clinical studies (14). Thiamine deficiency is also more prevalent in patients with advanced age, diuretic use and pregnancy (15). Although vitamin B1 deficiency is rare at present, it has attracted more and more attention as a risk factor for a variety of systemic diseases in recent years, as evidenced by the review of a large number of literature sources (16). Since the end of 2014, thiamine deficiency has re-emerged in the Pacific Islands, but the underlying cause of the re-emergence is still unknown, Therefore the study of vitamin B1 should not be overlooked (17). There is growing evidence that vitamin B1 supplementation can reverse CVD, diabetes, obesity, dyslipidemia, angina, myocardial infarction, and psychiatric disorders (18, 19). However, large population-based studies examining the association between vitamin B1 intake and cardiovascular disease and mortality are lacking. To address this gap, data was collected from the National Health and Nutrition Examination Survey (NHANES) from 1999 to 2018 and an analysis of large, nationally representative clinical studies was conducted to evaluate the association of dietary vitamin B1 intake with cardiovascular and all-cause mortality, and provide a theoretical basis for prevention.

## 2. Methods

### 2.1. Study population

The National Health and Nutrition Examination Survey (NHANES) is a large-scale, stratified, multi-stage sampling study conducted by the US Centers for Disease Control and Prevention (20). NHANES are widely used as large prospective cohorts with nationally representative samples through their association with follow-up mortality data (21). The NCHS Ethics Review Board approved all NHANES programs, and study members or their agents provided informed consent prior to participation (22). Detailed information about NHANES can be found at www.cdc.gov/nchs/nhanes/.

The data from 10 NHANES cycles from 1999 to 2018 was screened, involving 101,316 participants. Among these individuals, data with missing information on vitamin B1 intake (*n* = 38,611), as well as participants without documented cardiovascular diseases, all-cause death, and cardiovascular mortality (*n* = 27,186) were excluded. Confounding factors with missing values were also excluded (*n* = 7,561). Ultimately, a total of 27,958 participants were included in the analysis (Supplementary Figure S1).

### 2.2. Variables

Dietary data were obtained from the NHANES database, and all participants were interviewed about a dietary recall for two 24-h periods. The first recall interview was conducted at the NHANES Mobile Test Center, and the second was conducted by telephone 10 days later. Total daily amounts of all nutrients/food components were calculated, using the USDA Food and Nutrition Dietary Research Database and were input into the NHANES database (23). In the study, the mean intake of vitamin B1 was analyzed from two 24-h recalls. Cardiovascular diseases were identified depending on their answers to a questionnaire wherein the participants were asked if they had been diagnosed by a medical professional with coronary heart disease (24). Measurements for myocardial infarction and congestive heart failure were similar. Participants were considered to have high blood pressure if any of the following criteria were met: informed by a medical expert about their increased blood pressure, or were taking blood pressure medication and had a mean systolic blood pressure (SBP) of 140 mmHg or diastolic blood pressure (DBP) of 90 mmHg at the physical examination. Participants were considered to have diabetes if one of the following was true: an individual informed by a medical expert of their contracting of diabetes and was taking insulin, or taking diabetes medication to lower blood sugar (25).

The information regarding all-cause mortality and cardiovascular conditions was accessed in the 2019 NHANES Linked Mortality File (LMF), which is available for public use. This file contains data from previous surveys that include mortality factors and the status of the participants from 1999 to December 31st. 2019. The information from the death certificates was used to derive the Clinical Modification System codes I00-I78 to determine the mortality rate resulting from all-cause mortality and cardiovascular diseases by the International Classification of Diseases, 10th Revision. Further details of the participants regarding the mortality factors can be accessed at https://www.cdc.gov/nchs/data-linkage/mortality-public.htm (26).

Based on existing information relevant covariates such as age, sex, level of education (less than 9th grade, grades 9–11, high school graduate/GED or equivalent record of formal schooling, partial college or AA degree or above, college graduate or above), BMI, smoking history (never smoked, smoking history), drinking history (less or more than 12 cups/year), aspirin use, family poverty income ratio (PIR:<1.0; 1.0–3.0; >3.0), physical activity, diabetes status, total energy intake, and laboratory measures of blood lipids were included. The energy intake of adults was calculated using the USDA Food and Nutrient Database, available at https://www.ars.usda.gov/nea/bhnrc/fsrg, which was designed to provide an effective and accurate method to collect intakes for large-scale national surveys (27). Detailed information on dietary vitamin B1 intake and other variables can be accessed at www.cdc.gov/nchs/nhanes/.

### 2.3. Statistical analysis

The information from NHANES (1999–2018) was merged and extracted on R Studio 4.1.3 (R Foundation for Statistical Computing, Vienna, Austria). The GraphPad Prism 8.0 (GraphPad Software, San Diego, CA, United States), R (4.1.3) studio, and EmpowerStats^1^ were used to analyze the graphic design and data. The Mann–Whitney *U* test, a nonparametric test, or the t-test was utilized to compare baseline characteristics for continuous variables, whereas categorical variables were compared with the chi-square or Fisher test. The Multivariate logistic regression models were utilized to estimate the correlation between the intake of dietary vitamin B1 and cardiovascular diseases (hypertension, coronary heart disease, myocardial infarction, heart failure) in each quartile. The hazard ratio of the mortality rate of all-cause and cardiovascular conditions to the intake of vitamin B1 in the diet was analyzed using multivariate Cox regression analysis. In each quartile, the hazard ratios (HRs) and 95% confidence intervals (CIs) were displayed using the lowest quartile (Q1) as a reference. Age, sex, body mass index (BMI), smoking history, drinking history and blood lipid were taken into consideration when performing stratified analysis. A 3-bar restricted cubic spline was constructed to visualize the dose–response relation of vitamin B1 intake and the risk of incidence of cardiovascular diseases along with all-cause, and cardiovascular mortality. To test the robustness of our findings, we conducted sensitivity analyses excluding the participants who died during the first year of follow-up (28). *p*-value <0.05 was regarded as statistically significant when all data were examined utilizing R studio v4.1.3.

## 3. Results

### 3.1. Characteristics of participants

The mean age of the 27,958 participating individuals was 50.10 ± 17.61 years of which 49.50% were male (*n* = 13,842). The results showed that relative to quartile four (high intake) there was a higher likelihood of female participants being included in quartile one (low intake). Education level showed that 52.96% of participants had an educational background of university, AA education, college graduate or above. There were 3,887 deaths, including 974 deaths caused by cardiovascular diseases. All the baseline characteristics of the participating individuals were assessed (Table 1).

**Table 1:** Baseline characteristics of total participants and stratification by the quartile of vitamin B1 intake.

Characteristic	Total subjects	Vitamin B1 Intake Quartile, mg/day				
		Q1 (Low) (0–1.04)	Q2 (1.04–1.43)	Q3 (1.43–1.94)	Q4 (High) (1.94–15.64)	*p*-value
*Number*	27,958	6,979	6,994	6,989	6,996	
*Median intake*	1.43	0.82	1.24	1.65	2.40	
*Average (SD)*	1.58 ± 0.81	0.78 ± 0.20	1.24 ± 0.11	1.66 ± 0.14	2.65 ± 0.81	
*Demographics*						
*Age (years old)*	50.10 ± 17.61	51.44 ± 17.74	51.50 ± 17.81	50.36 ± 17.53	47.10 ± 17.00	<0.001
<50	13,821 (49.43)	3,186 (45.64)	3,214 (45.95)	3,444 (49.28)	3,979 (56.88)	
≥50	14,137 (50.57)	3,794 (54.36)	3,781 (54.05)	3,545 (50.72)	3,017 (43.12)	
*Sex, n (%)*						<0.001
Male	13,840 (49.50)	2,254 (32.30)	2,902 (41.49)	3,622 (51.82)	5,062 (72.36)	
Female	14,118 (50.50)	4,725 (67.70)	4,092 (58.51)	3,367 (48.18)	1934 (27.64)	
*Level of education, n (%)*						<0.001
Less than 9th grade	2,758 (9.86)	934 (13.38)	760 (10.87)	577 (8.26)	487 (6.96)	
9-11th grade	3,935 (14.07)	1,177 (16.86)	979 (14.00)	872 (12.48)	907 (12.96)	
High school graduate/ GED or equivalent	6,457 (23.10)	1,642 (23.53)	1,638 (23.42)	1,621 (23.19)	1,556 (22.24)	
Some college or AA degree	8,220 (29.40)	2024 (29.00)	2068 (29.57)	2065 (29.55)	2063 (29.49)	
College graduate or above	6,588 (23.56)	1,202 (17.22)	1,549 (22.15)	1854 (26.53)	1983 (28.34)	
*BMI (kg/m^2^)*						<0.001
<25	7,947 (28.42)	1862 (26.68)	1902 (27.19)	1999 (28.60)	2,184 (31.22)	
25–30	9,434 (33.74)	2,276 (32.61)	2,368 (33.86)	2,360 (33.77)	2,430 (34.73)	
≥30	10,577 (37.83)	2,841 (40.71)	2,724 (38.95)	2,630 (37.63)	2,382 (34.05)	
*Smoking history,* *n (%)*						<0.001
Never	15,037 (53.78)	3,683 (52.77)	3,817 (54.58)	3,875 (55.44)	3,662 (52.34)	
smoker	12,921 (46.22)	3,296 (47.23)	3,177 (45.42)	3,114 (44.56)	3,334 (47.66)	
*Drinking history, n (%)*						0.012
<12	8,599 (30.76)	2,574 (36.88)	2,259 (32.30)	2059 (29.46)	1707 (24.40)	
≥12	19,359 (69.24)	4,405 (63.12)	4,735 (67.70)	4,930 (70.54)	5,289 (75.60)	
*Aspirin use, n (%)*						<0.001
No	27,602 (98.73)	6,873 (98.48)	6,896 (98.60)	6,902 (98.76)	6,931 (99.07)	
Yes	356 (1.27)	106 (1.52)	98 (1.40)	87 (1.24)	65 (0.93)	
*Poverty to income ratio, n (%)*						<0.001
0.0–1.0	5,377 (19.23)	1,665 (23.86)	1,288 (18.42)	1,208 (17.29)	1,216 (17.38)	
1.01–3.0	11,781 (42.14)	3,087 (44.23)	3,060 (43.75)	2,882 (41.24)	2,752 (39.34)	
>3.0	10,799 (38.63)	2,227(31.91)	2,646 (37.83)	2,898 (41.47)	3,028 (43.28)	
*Physical activity* *(MET-min/wk), n (%)*						<0.001
<500	12,453 (44.54)	3,606 (51.66)	3,213 (45.94)	2,970 (42.50)	2,664 (38.08)	
500–999	3,140 (11.23)	766 (10.97)	798 (11.41)	843 (12.06)	733 (10.48)	
≥1,000	12,365 (44.23)	2,607 (37.36)	2,983 (42.65)	3,176 (45.44)	3,599 (51.44)	
*Total energy intake* *(kcal/d), (SD)*	2110.92 ± 992.86	1400.98 ± 588.07	1868.77 ± 649.40	2236.58 ± 760.23	2935.67 ± 1156.84	<0.001
*Laboratory measurements*						
TC (mmol/L)	5.05 ± 1.08	5.12 ± 1.09	5.08 ± 1.09	5.03 ± 1.08	4.97 ± 1.07	<0.001
TG (mmol/L)	1.73 ± 1.54	1.60 ± 1.16	1.73 ± 1.61	1.76 ± 1.71	1.81 ± 1.59	<0.001
HDL (mmol/L)	1.35 ± 0.41	1.39 ± 0.43	1.38 ± 0.41	1.35 ± 0.40	1.29 ± 0.38	<0.001
*Diseases*						
*HTN, n (%)*						<0.001
No	15,915 (56.92)	3,703 (53.05)	3,931 (56.20)	4,044 (57.86)	4,392 (62.78)	
Yes	12,043 (43.08)	3,277 (46.95)	3,064 (43.80)	2,945 (42.14)	2,604 (37.22)	
*DM, n (%)*						<0.001
No	20,883 (74.69)	5,136 (73.58)	5,089 (72.75)	5,185 (74.19)	5,473 (78.23)	
Yes	4,960 (17.74)	1,348 (19.31)	1,377 (19.69)	1,230 (17.60)	1,006 (14.38)	
IFG + IGT	2,116 (7.57)	496 (7.11)	529 (7.56)	574 (8.21)	517 (7.39)	
*CHD, n (%)*						0.236
No	26,756 (95.69)	6,685 (95.77)	6,672 (95.38)	6,679 (95.56)	6,720 (96.05)	
Yes	1,204 (4.31)	295 (4.23)	323 (4.62)	310 (4.44)	276 (3.95)	
*MI, n (%)*						<0.001
No	26,763 (95.73)	6,637 (95.09)	6,679 (95.48)	6,701 (95.88)	6,748 (96.46)	
Yes	1,195 (4.27)	343 (4.91)	316 (4.52)	288 (4.12)	248 (3.54)	
*HF, n (%)*						<0.001
No	27,073 (96.83)	6,703 (96.03)	6,733 (96.25)	6,795 (97.22)	6,844 (97.83)	
Yes	885 (3.17)	277 (3.97)	262 (3.75)	194 (2.78)	152 (2.17)	
*Mortality risk*						
*ACM, n(%)*						<0.001
No	24,071 (86.10)	5,805 (83.18)	6,032 (86.25)	6,041 (86.44)	6,193 (88.52)	
Yes	3,887 (13.90)	1,174 (16.82)	962 (13.75)	948 (13.56)	803 (11.48)	
*CVDM, n (%)*						<0.001
No	26,984 (96.52)	6,677 (95.67)	6,741 (96.38)	6,747 (96.54)	6,819 (97.47)	
Yes	974 (3.48)	302 (4.33)	253 (3.62)	242 (3.46)	177 (2.53)	

BMI, Body Mass Index; TC, Total Cholesterol; TG, Triglyceride; HDL, High Density Lipoprotein; HTN, Hypertension; DM, Diabetes Mellitus; CHD, Coronary Heart Disease; MI, Myocardial Infarction; HF, Heart Failure; ACM, All-cause Mortality; CVDM, Cardiovascular Mortality.

### 3.2. Prevalence of cardiovascular diseases and trends of all-cause and cardiovascular mortality

**Figure 1:**
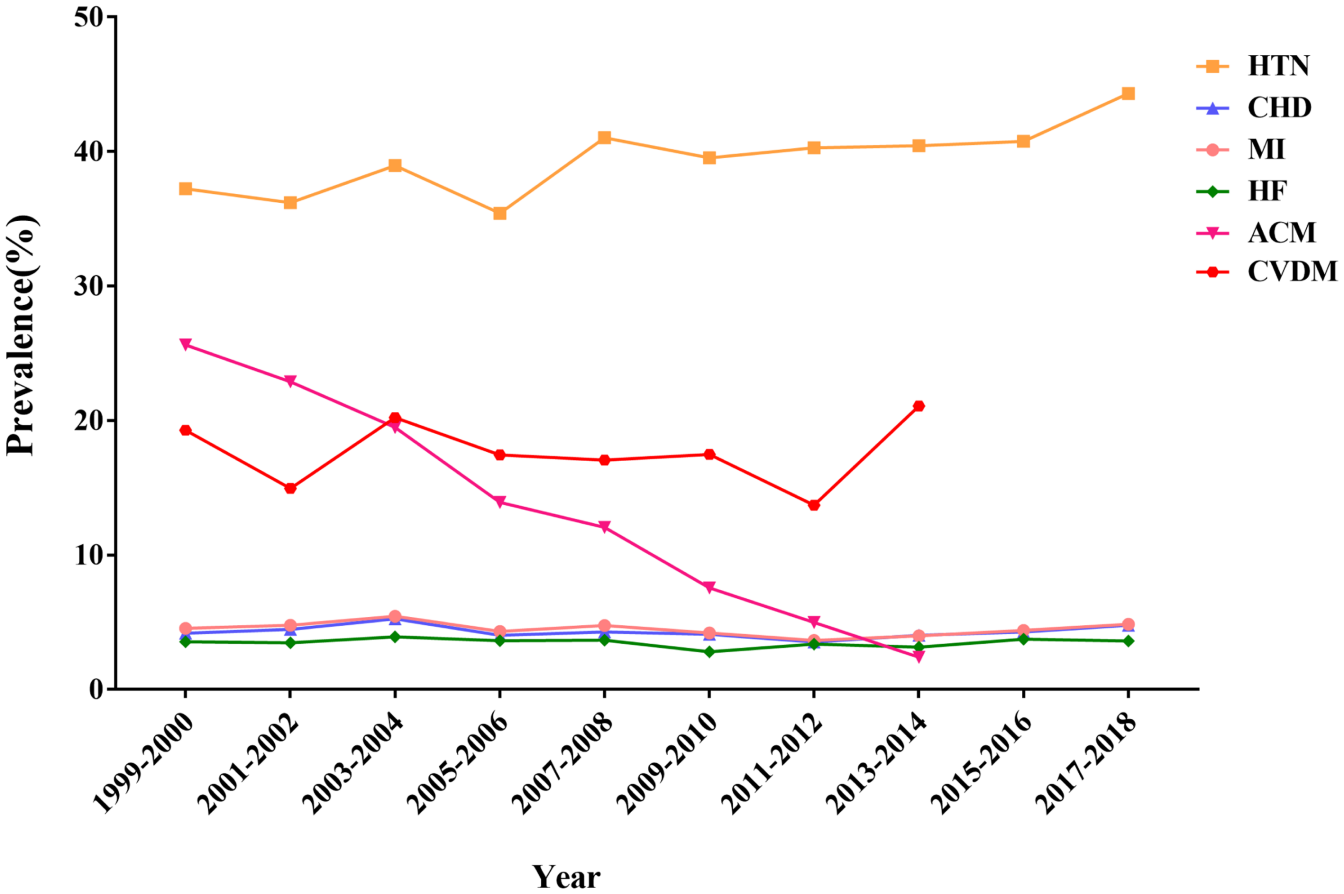
Trends in prevalence of cardiovascular diseases and all-cause and cardiovascular mortality among adults in the United States, 1999–2018.

The NHANES database highlights a concerning trend in the prevalence of cardiovascular diseases in United States adults from 1999 to 2018, Hypertension saw a significant increase from 37.2% in 1999–2000 to 44.3% in 2017–2018. Additionally, within the aforementioned time range, coronary heart disease rose from 4.2 to 4.8% and myocardial infarction increased from 4.5 to 4.9%. The prevalence of heart failure did not increase significantly. However, the all-cause mortality rate decreased significantly from 25.6% in 1999–2000 to 2.4% in 2017–2018, and the cardiovascular mortality rate increased from 19.3 to 21.1%. Figure 1 shows the trends in the prevalence of various types of cardiovascular diseases and cardiovascular mortality as well as all-cause mortality in the United States adult population.

### 3.3. Effects of various factors on cardiovascular diseases, all-cause and cardiovascular mortality by univariate analysis

Most parameters were correlated with cardiovascular diseases, all-cause, and cardiovascular mortality. The risk of hypertension, coronary heart disease, myocardial infarction, heart failure, all- cause mortality, and cardiovascular mortality (95% confidence interval; *p*-value) among participants aged ≥50 years was 6.76 (6.41, 7.13; *p* < 0.001), 16.62 (13.08, 21.12; *p* < 0.001), 10.36 (8.50, 12.63; *p* < 0.001), 8.76 (7.06, 10.87; *p* < 0.001), 10.99 (9.91, 12.20; *p* < 0.001) and 13.92 (11.08, 17.48; *p* < 0.001), respectively compared with participants younger than 50 years. Except for hypertension, the odds ratios or hazard ratios (95% confidence interval; *p*-value) for this correlation were 0.44 (0.39, 0.50; *p* < 0.001), 0.47 (0.41, 0.53; *p* < 0.001), 0.72 (0.63, 0.82; *p* < 0.001), 0.74 (0.70, 0.79; *p* < 0.001) and 0.66 (0.58, 0.75; *p* < 0.001) for female relative to male, respectively. With the exception of 9-11th grade, all other parameters were associated with cardiovascular diseases, all-cause and cardiovascular mortality (Tables 2, 3).

**Table 2:** Effects of various factors on cardiovascular diseases by univariate analysis.

Variables	OR(95%CI) *p*-value			
	HTN	CHD	MI	HF
*Age(years old)*				
<50	1.0	1.0	1.0	1.0
≥50	6.76 (6.41, 7.13) <0.001	16.62 (13.08, 21.12) <0.001	10.36 (8.50, 12.63) <0.001	8.76 (7.06, 10.87) <0.001
*Sex, n (%)*				
Male	1.0	1.0	1.0	1.0
Female	1.00 (0.96, 1.05) 0.854	0.44 (0.39, 0.50) <0.001	0.47 (0.41, 0.53) <0.001	0.72 (0.63, 0.82) <0.001
*Level of education, n (%)*				
Less than 9th grade	1.0	1.0	1.0	1.0
9-11th grade	0.79 (0.71, 0.87) <0.001	0.79 (0.64, 0.98) 0.029	0.72 (0.58, 0.88) 0.001	0.84 (0.67, 1.05) 0.123
High school graduate/ GED or equivalent	0.77 (0.70, 0.84) <0.001	0.73 (0.60, 0.89) 0.002	0.66 (0.55, 0.79) <0.001	0.65 (0.52, 0.80) <0.001
Some college or AA degree	0.65 (0.61, 0.71) <0.001	0.59 (0.49, 0.72) <0.001	0.51 (0.43, 0.62) <0.001	0.54 (0.44, 0.67) <0.001
College graduate or above	0.50 (0.45, 0.54) <0.001	0.57 (0.46, 0.69) <0.001	0.39 (0.32, 0.48) <0.001	0.26 (0.20, 0.34) <0.001
*BMI (kg/m^2^)*				
<25	1.0	1.0	1.0	1.0
25–30	1.86 (1.75, 1.99) <0.001	1.45 (1.24, 1.70) <0.001	1.32 (1.12, 1.56) <0.001	1.36 (1.12, 1.66) 0.002
≥30	3.05 (2.87, 3.25) <0.001	1.62 (1.39, 1.89) <0.001	1.73 (1.48, 2.01) <0.001	2.20 (1.84, 2.64) <0.001
*Smoking history, n (%)*				
No	1.0	1.0	1.0	1.0
Yes	1.33 (1.27, 1.40) <0.001	2.19 (1.94, 2.47) <0.001	2.40 (2.12, 2.71) <0.001	2.00 (1.74, 2.30) <0.001
*Drinking history, n (%)*				
No	1.0	1.0	1.0	1.0
Yes	0.70 (0.66, 0.73) <0.001	0.87 (0.77, 0.98) 0.023	0.95 (0.84, 1.07) 0.390	0.69 (0.60, 0.80) <0.001
*Diabetes, n (%)*				
No	1.0	1.0	1.0	1.0
Yes	4.85 (4.52, 5.18) <0.001	4.30 (3.80, 4.86) <0.001	3.95 (3.49, 4.46) <0.001	4.65 (4.04, 5.36) <0.001
IGT + IFG	2.18 (1.99, 2.38) <0.001	2.08 (1.69, 2.55) <0.001	1.71 (1.38, 2.13) <0.001	1.69 (1.30, 2.20) <0.001
*Aspirin use, n (%)*				
No	1.0	1.0	1.0	1.0
Yes	5.61 (4.31, 7.30) <0.001	5.50 (4.23, 7.27) <0.001	5.50 (4.20, 7.21) <0.001	4.49 (3.25, 6.20) <0.001
*Poverty to income ratio, n (%)*				
0.0–1.0	1.0	1.0	1.0	1.0
1.01–3.0	1.14 (1.07, 1.21) 0.001	1.21 (1.03, 1.43) 0.020	0.92 (0.79, 1.06) 0.258	0.97 (0.83, 1.15) 0.752
>3.0	0.92 (0.86, 0.99) 0.020	1.05 (0.89, 1.24) 0.587	0.59 (0.50, 0.70) <0.001	0.43 (0.35, 0.52) <0.001
*Physical activity (MET-min/wk), n (%)*				
<500	1.0	1.0	1.0	1.0
500–999	0.70 (0.65, 0.76) <0.001	0.88 (0.73, 1.06) 0.174	0.74 (0.61, 0.89) 0.002	0.50 (0.39, 0.64) <0.001
≥1,000	0.58 (0.55, 0.61) <0.001	0.59 (0.52, 0.67) <0.001	0.51 (0.45, 0.58) <0.001	0.37 (0.32, 0.43) <0.001
*Total energy intake (kcal/d), (SD)*	1.00 (1.00,1.00) <0.001	1.00 1.00,1.00) <0.001	1.00 (1.00,1.00) <0.001	1.00(1.00,1.00) <0.001
TC(mmol/L)	1.08 (1.05, 1.10) <0.001	0.60 (0.56, 0.63) <0.001	0.66 (0.63, 0.71) <0.001	0.68 (0.64, 0.73) <0.001
TG(mmol/L)	1.18 (1.15, 1.20) <0.001	1.06 (1.03, 1.08) <0.001	1.05 (1.02, 1.08) <0.001	1.06 (1.03, 1.09) <0.001
HDL(mmol/L)	0.92 (0.87, 0.97) 0.005	0.42 (0.36, 0.50) <0.001	0.50 (0.43, 0.59) <0.001	0.45 (0.38, 0.55) <0.001

**Table 3:** Effects of various factors on all cause mortality and cardiovascular mortality by univariate analysis.

Variables	HR(95%CI) *p*-value	
	ACM	CVD
*Age(years old)*		
<50	1.0	1.0
≥50	10.99 (9.91, 12.20) <0.001	13.92 (11.08, 17.48) <0.001
*Sex, n (%)*		
Male	1.0	1.0
Female	0.74 (0.70, 0.79) <0.001	0.66 (0.58, 0.75) <0.001
*Level of education, n (%)*		
Less than 9th grade	1.0	1.0
9-11th grade	0.77 (0.70, 0.86) <0.001	0.73 (0.60, 0.90) 0.003
High school graduate/ GED or equivalent	0.69 (0.63, 0.76) <0.001	0.68 (0.56, 0.82) <0.001
Some college or AA degree	0.49 (0.45, 0.55) <0.001	0.46 (0.37, 0.56) <0.001
College graduate or above	0.40 (0.35, 0.44) <0.001	0.39 (0.32, 0.49) <0.001
*BMI (kg/m^2^)*		
<25	1.0	1.0
25–30	1.02 (0.94, 1.10) 0.694	1.32 (1.12, 1.55) 0.001
≥30	0.97 (0.90, 1.05) 0.433	1.24 (1.05, 1.45) 0.011
*Smoking history, n (%)*		
No	1.0	1.0
Yes	1.81 (1.69, 1.93) <0.001	1.64 (1.45, 1.87) <0.001
*Drinking history, n (%)*		
No	1.0	1.0
Yes	0.79 (0.74, 0.85) <0.001	0.73 (0.64, 0.83) <0.001
*Diabetes, n (%)*		
No	1.0	1.0
Yes	3.09 (2.88, 3.31) <0.001	3.70 (3.23, 4.24) <0.001
IGT + IFG	1.97 (1.76, 2.20) <0.001	2.05 (1.63, 2.58) <0.001
*Aspirin use, n (%)*		
No	1.0	1.0
Yes	2.88 (2.40, 3.45) <0.001	3.10 (2.18, 4.41) <0.001
*Poverty to income ratio, n (%)*		
0.0–1.0	1.0	1.0
1.01–3.0	1.18 (1.09, 1.28) <0.001	1.23 (1.04, 1.45) 0.015
>3.0	0.65 (0.59, 0.71) <0.001	0.65 (0.54, 0.78) <0.001
*Physical activity (MET-min/wk), n (%)*		
<500	1.0	1.0
500–999	0.62 (0.55, 0.68) <0.001	0.68 (0.56, 0.83) <0.001
≥1,000	0.53 (0.49, 0.57) <0.001	0.44 (0.38, 0.52) <0.001
*Total energy intake (kcal/d), (SD)*	1.00(1.00,1.00) <0.001	1.00(1.00,1.00) <0.001
TC(mmol/L)	0.98 (0.96, 1.01) 0.290	0.96 (0.90, 1.02) 0.187
TG(mmol/L)	1.03 (1.01, 1.04) <0.001	1.02 (0.98, 1.05) 0.303
HDL(mmol/L)	1.10 (1.02, 1.19) 0.012	0.88 (0.75, 1.04) 0.138

### 3.4. Correlation analysis of vitamin B1 intake with cardiovascular diseases, all-cause, and cardiovascular mortality

**Figure 2:**
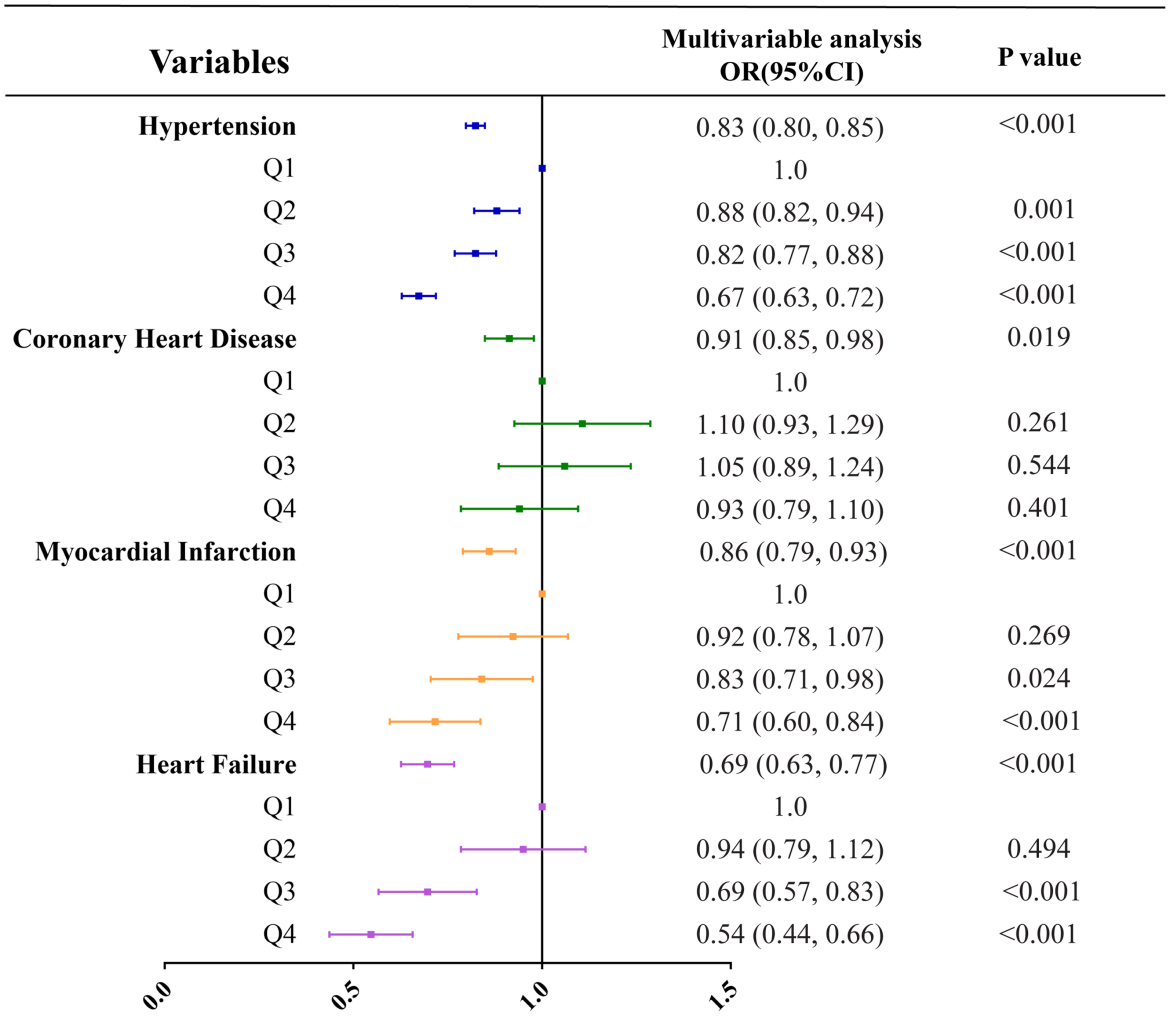
Correlation analysis between vitamin B1 and various types of cardiovascular diseases under unadjusted models.

**Figure 3:**
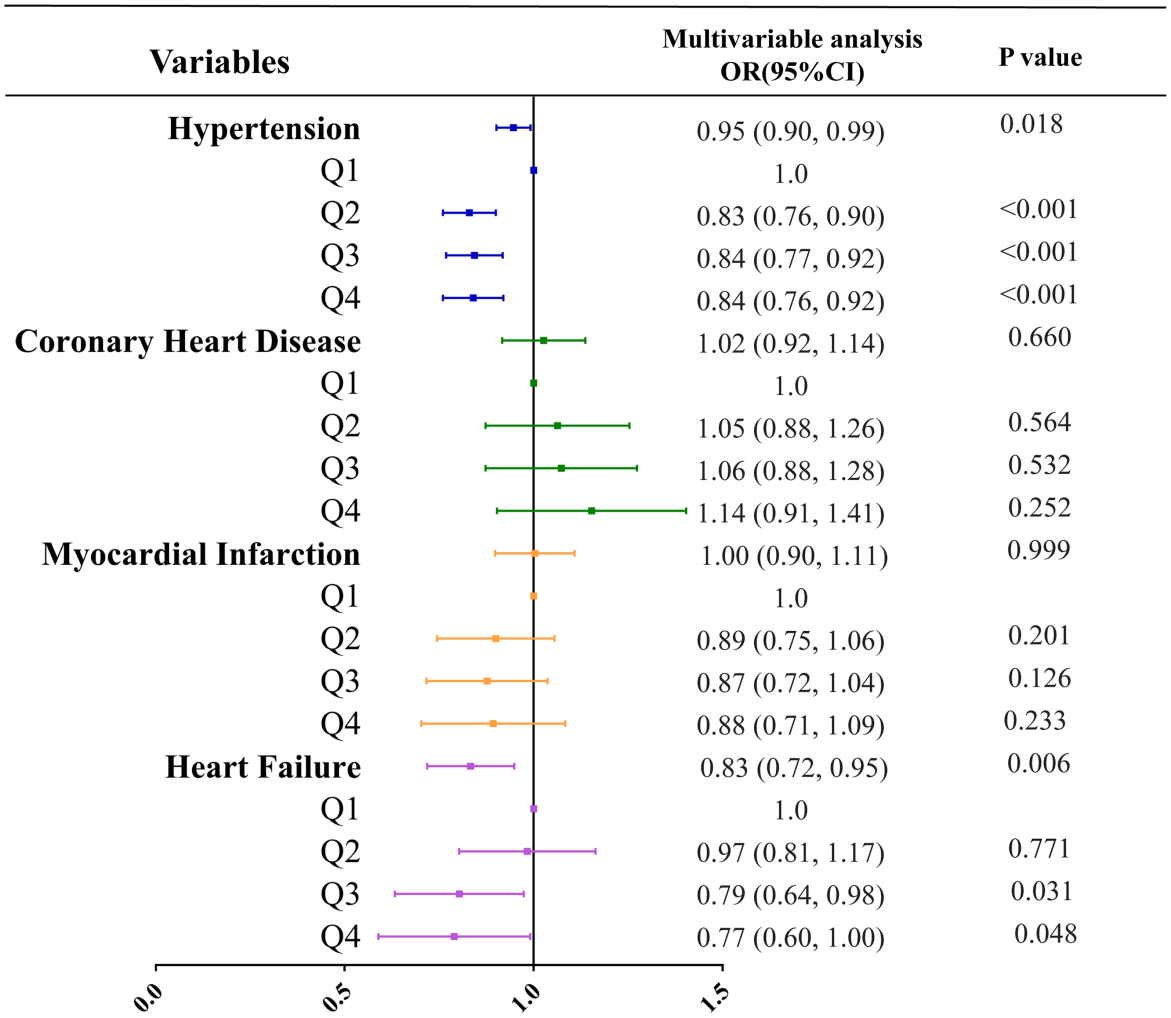
Correlation analysis between vitamin B1 and various types of cardiovascular diseases under all covariates adjusted models.

**Figure 4:**
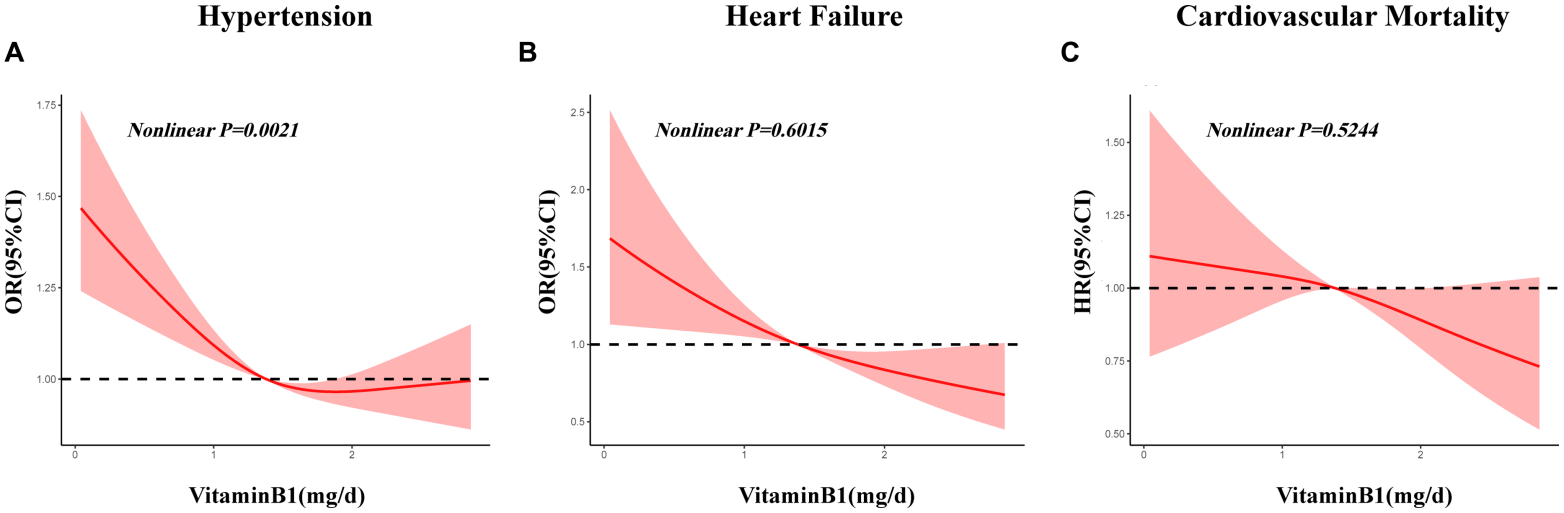
Restricted cubic spline analysis of associations between vitamin B1 intake and hypertension, heart failure and cardiovascular mortality. **(A)** Restricted cubic spline analysis of association between vitamin B1 intake and hypertension; **(B)** Restricted cubic spline analysis of association between vitamin B1 intake and heart failure; **(C)** Restricted cubic spline analysis of association between vitamin B1 intake and cardiovascular mortality.

Three multivariate logistic regression models were developed: Model 1, unadjusted; Model 2, adjusted for age, sex, and level of education; Model 3, adjusted for all covariates listed in Table 1. In unadjusted models, vitamin B1 intake was inversely correlated with all types of cardiovascular diseases, and the respective odds ratios for hypertension, coronary heart disease, myocardial infarction and heart failure were 0.83 (0.80, 0.85; *p* < 0.001), 0.91 (0.85, 0.98; *p* = 0.019), 0.86 (0.79, 0.93; *p* < 0.001) and 0.69 (0.63, 0.77; *p* < 0.001) (Table 4; Figure 2). In the model adjusted for all covariates, vitamin B1 intake was significantly negatively associated with hypertension and heart failure, with odds ratios of 0.95 (0.90, 0.99; *p* = 0.018) and 0.83 (0.72, 0.95; *p =* 0.006), respectively, but not with coronary heart disease and myocardial infarction (Table 4; Figure 3). Restricted cubic spline analysis showed a nonlinear inverse association between vitamin B1 intake and hypertension and a linear association with heart failure (Figure 4).

**Table 4:** Multivariable associations of vitamin B1 with cardiovascular diseases.

	Model 1	Model 2	Model 3
	OR(95% CI) *p* value	OR(95% CI) *p* value	OR(95% CI) *p* value
*HTN*			
Vitamin B1 (mg/d)	**0.83 (0.80, 0.85) < 0.001**	**0.94 (0.91, 0.98) 0.002**	**0.95 (0.90, 0.99) 0.018**
Vitamin B1 (mg/d) quartiles			
Q1	**1.0**	**1.0**	1.0
Q2	**0.88 (0.82, 0.94) 0.001**	**0.85 (0.79, 0.92) < 0.001**	**0.83 (0.76, 0.90) < 0.001**
Q3	**0.82 (0.77, 0.88) < 0.001**	**0.87 (0.80, 0.94) < 0.001**	**0.84 (0.77, 0.92) < 0.001**
Q4	**0.67 (0.63, 0.72) < 0.001**	**0.85 (0.78, 0.92) < 0.001**	**0.84 (0.76, 0.92) < 0.001**
*CHD*			
Vitamin B1 (mg/d)	**0.91 (0.85, 0.98) 0.019**	0.94 (0.86, 1.02) 0.134	1.02 (0.92, 1.14) 0.660
Vitamin B1 (mg/d) quartiles			
Q1	1.0	1.0	1.0
Q2	1.10 (0.93, 1.29) 0.261	1.01 (0.86, 1.20) 0.869	1.05 (0.88, 1.26) 0.564
Q3	1.05 (0.89, 1.24) 0.544	0.96 (0.80, 1.14) 0.604	1.06 (0.88, 1.28) 0.532
Q4	0.93 (0.79, 1.10) 0.401	0.94 (0.78, 1.12) 0.487	1.14 (0.91, 1.41) 0.252
*MI*			
Vitamin B1 (mg/d)	**0.86 (0.79, 0.93) < 0.001**	**0.88 (0.81, 0.96) 0.004**	1.00 (0.90, 1.11) 0.999
Vitamin B1 (mg/d) quartiles			
Q1	1.0	1.0	1.0
Q2	0.92 (0.78, 1.07) 0.269	0.85 (0.72, 1.00) 0.054	0.89 (0.75, 1.06) 0.201
Q3	**0.83 (0.71, 0.98) 0.024**	**0.76 (0.65, 0.91)0.002**	0.87 (0.72, 1.04) 0.126
Q4	**0.71 (0.60, 0.84) < 0.001**	**0.71 (0.59, 0.85) < 0.001**	0.88 (0.71, 1.09) 0.233
*HF*			
Vitamin B1 (mg/d)	**0.69 (0.63, 0.77) < 0.001**	**0.76 (0.68, 0.85) < 0.001**	**0.83 (0.72, 0.95) 0.006**
Vitamin B1 (mg/d) quartiles			
Q1	1.0	1.0	1.0
Q2	0.94 (0.79, 1.12) 0.494	0.93 (0.78, 1.11) 0.403	0.97 (0.81, 1.17) 0.771
Q3	**0.69 (0.57, 0.83) < 0.001**	**0.71 (0.58, 0.86) < 0.001**	**0.79 (0.64, 0.98) 0.031**
Q4	**0.54 (0.44, 0.66) < 0.001**	**0.64 (0.51, 0.79) < 0.001**	**0.77 (0.60, 1.00) 0.048**

Model 1: No adjustments made for confounding factors.

Model 2: Adjustments made for age, sex, level of education.

Model 3: Adjustments made for age, sex, level of education, BMI, smoking history, drinking history, aspirin use, diabetes mellitus, poverty to income ratio, physical activity, Total energy intake, TC, TG, HDL.

Bold values represent significant associations.

**Figure 5:**
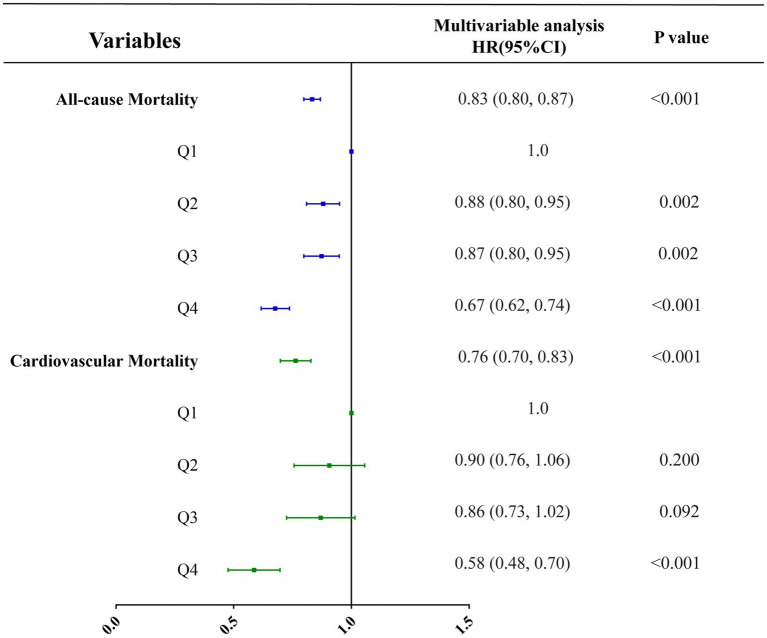
Correlation analysis between vitamin B1 and all-cause mortality and cardiovascular mortality under unadjusted models.

**Figure 6:**
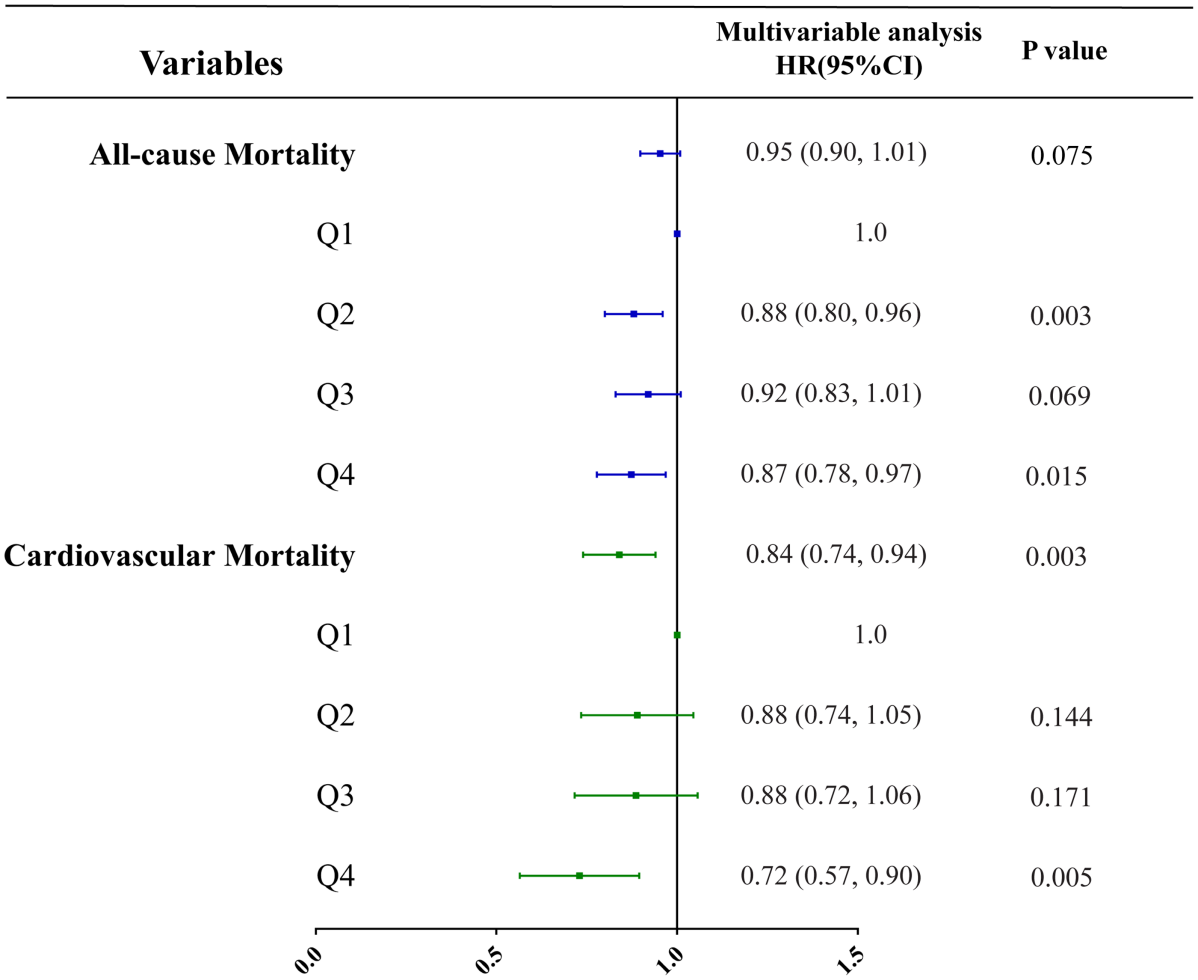
Correlation analysis between vitamin B1 and all-cause mortality and cardiovascular mortality under all covariates adjusted models.

Three multivariate cox regression models were developed: Model 1, unadjusted; Model 2, adjusted for age, sex, and level of education; Model 3, adjusted for all covariates listed in Table 1. In unadjusted models, vitamin B1 intake was inversely correlated with all-cause mortality and cardiovascular mortality, with hazard ratios of 0.83 (0.80, 0.87; *p* < 0.001) and 0.76 (0.70, 0.83; *p* < 0.001), respectively (Table 5; Figure 5). In the model adjusted for all covariates, vitamin B1 intake was significantly negatively associated with cardiovascular mortality, with a hazard ratio of 0.84 (0.74, 0.94; *p* = 0.003) (Table 5; Figure 6). Restricted cubic spline analysis showed a linear association with cardiovascular mortality and the dose–response correlation showed that the prevalence of cardiovascular diseases and mortality decreased with increasing vitamin B1 intake (Figure 4).

**Table 5:** Multivariable associations of vitamin B1 with all-cause mortality and cardiovascular mortality.

	Model 1 HR(95% CI) *p* value	Model 2 HR(95% CI) *p* value	Model 3 HR(95% CI) *p* value
*ACM*			
Vitamin B1 (mg/d)	**0.83 (0.80, 0.87) < 0.001**	**0.95 (0.91, 0.99) 0.019**	0.95 (0.90, 1.01) 0.075
Vitamin B1 (mg/d) quartiles			
Q1	**1.0**	1.0	1.0
Q2	**0.88 (0.80, 0.95) 0.002**	**0.88 (0.81, 0.96) 0.004**	**0.88 (0.80, 0.96) 0.003**
Q3	**0.87 (0.80, 0.95) 0.002**	**0.90 (0.82, 0.98) 0.019**	0.92 (0.83, 1.01) 0.069
Q4	**0.67 (0.62, 0.74) < 0.001**	**0.85 (0.78, 0.94) 0.001**	0.87 (0.78, 0.97) 0.015
*CVDM*			
Vitamin B1 (mg/d)	**0.76 (0.70, 0.83) < 0.001**	**0.85 (0.77, 0.93) < 0.001**	**0.84 (0.74, 0.94) 0.003**
Vitamin B1 (mg/d) quartiles			
Q1	1.0	1.0	1.0
Q2	0.90 (0.76, 1.06) 0.200	0.89 (0.75, 1.05) 0.167	0.88 (0.74, 1.05) 0.144
Q3	0.86 (0.73, 1.02) 0.092	0.86 (0.72, 1.02) 0.093	0.88 (0.72, 1.06) 0.171
Q4	**0.58 (0.48, 0.70) < 0.001**	**0.71 (0.58, 0.86) < 0.001**	**0.72 (0.57, 0.90) 0.005**

Model 1: No adjustments made for confounding factors.

Model 2: Adjustments made for age, sex, level of education.

Model 3: Adjustments made for age, sex, level of education, BMI, smoking history, drinking history, aspirin use, diabetes mellitus, poverty to income ratio, physical activity, Total energy intake, TC, TG, HDL.

Bold values represent significant associations.

### 3.5. Subgroup and sensitivity analyses

To determine whether the association between vitamin B1 intake and cardiovascular disease, all-cause mortality, and cardiovascular mortality varied by age, sex, BMI, smoking history, drinking history and blood lipid profile, stratified analyses were performed. When stratified by age, vitamin B1 intake was more significantly associated with hypertension and heart failure in patients aged ≥50 years, with odds ratios of 0.94 (0.91, 0.98; *p =* 0.002) and 0.79 (0.69, 0.91; *p* = 0.001), respectively(Supplementary Table S1). Similarly, the subgroups including males (Supplementary Table S2), those with a 25 ≤ BMI < 30 (Supplementary Table S3), smokers (Supplementary Table S4), drinkers (Supplementary Table S5), and those with dyslipidemia (Supplementary Table S6) were more strongly associated with hypertension, heart failure and mortality. The correlations of vitamin B1 intake in Q4 were higher than those in Q1. In the sensitivity analysis, after further adjustment for energy intake at baseline, these associations remained similar and statistically significant (Supplementary Table S7).

## 4. Discussion

In a nationally representative sample of US adults, vitamin B1 intake were associated with risk for hypertension, heart failure and cardiovascular mortality, whereas were not associated with coronary heart disease, myocardial infarction and all-cause mortality. The study also provided trends in vitamin B1 intake, prevalence of cardiovascular-related disease, and cardiovascular and all-cause mortality among U.S. adults from 1999 to 2018. From 1999 to 2018, the all-cause mortality of adults in the United States showed a significant downward trend, but the mortality and prevalence of cardiovascular disease showed an upward trend.

This study found a linear negative correlation between vitamin B1 intake and heart failure and cardiovascular mortality. Using restricted spline analysis, vitamin B1 intake was non-linearly associated with hypertension. In addition, the correlation was more significant in the high-intake group of vitamin B1 compared with the low-intake group. The findings of this research were consistent with those of a previous study that reported that daily intake of thiamine could reverse the risk of hypertension (OR 0.95; 95% CI 0.90, 0.99), myocardial infarction or angina pectoris (OR 0.84; 95% CI 0.74, 0.95), type 2 diabetes (OR 0.86; 95% CI 0.81, 0.93), depression (OR 0.90; 95% CI 0.83, 0.97) and dyslipidemia (OR 0.90; 95% CI 0.86, 0.95) (18). Vitamin B1 could be a potential low-cost and effective intervention, which could improve cardiac function (29, 30) and hemodynamic characteristics (31), as well as reduce systemic vascular resistance (32). In a previous study, thiamine, mediated by glucose and insulin, had a protective effect on the propagation of human smooth muscle cells of the inferior artery, which were reported to be crucial in the formation of atherosclerotic plaque (33). A trial conducted on 30 hyperglycemic participants (10 subjects each grouped into healthy, impaired glucose tolerance, and T2D) by Arora et al. examined the impact on endothelium-dependent vasodilation after using 100 mg of intravenous thiamine. All the groups depicted improvement. Arora et al. suggested that since the stability of endothelial function was closely correlated to the occurrence of coronary atherosclerotic heart disease, therefore, thiamine should be administered regularly to enhance endothelial function and slow the development of atherosclerosis (34). In patients with stable HF, several researchers had studied whether thiamine supplementation could improve their cardiac health. The randomized, double-blind controlled trial conducted by Schoenenberger et al. (35) demonstrated that the left ventricular ejection fraction (LVEF) was 3.30% (95% CI 0.63–5.97%) higher in patients taking thiamine than in those receiving a placebo. Furthermore, Shimon et al. (36) found in a double-blind randomized study that LVEF was increased by 2.20% in the group taking thiamine than in the placebo group, but their improvement was not statistically significant. Dinicolantonio et al. (15) performed mate analysis and the data indicated that compared with the placebo group, thiamine supplementation significantly improved LVEF (3.28, 95% CI 0.64–5.93%), which was important for the treatment of heart failure and prevention of recurrence. Moreover, it might be correlated to the improvement of energy metabolism in cardiomyocytes. Additionally, Jain et al. (37) performed a meta-analysis and depicted that deficiency of thiamine was more prevalent in patients with HF than in healthy individuals. Although thiamine supplementation is generally not recommended in patients with systolic heart failure, studies have shown that it can have a positive effect on LVEF in patients with systolic heart failure. Alattas et al. (38) found that supplementation of thiamine (100 mg/d) for 6 months could significantly control and alleviate cardiovascular complications in diabetic patients. Common risk factors for thiamine deficiency in patients with heart failure include malnutrition, increased metabolic status, the severity of heart failure, and the use of diuretics (39). In a meta-analysis, LVEF was significantly improved by 3.28% (95% CI 0.64–5.93) in 34 patients treated with thiamine but the meta-analysis was limited by the quality of the original study and the small sample size (39). These findings suggested that thiamine had a protective effect against vascular diseases, of which heart failure might be more prominent, and are consistent with the results of the present study.

It is worth noting that the associations of interest observed in the aforementioned studies were similar to those in the current analyses. However, the current study was based on a much larger sample size, providing greater statistical power for detecting modest associations. Furthermore, the current study conducted numerous subgroup analyses, exploring the impact of vitamin B1 intake on different populations. The findings indicated that vitamin B1 (thiamine) was more significantly correlated with heart failure in elderly people (whose age ≥ 50 years old). This could be due to the increased risk of micronutrient deficiency in the elderly caused by pathophysiological changes (40). The elderly are more susceptible to thiamine deficiency due to nutritional challenges and various potential comorbidities (41). Prior systematic reviews and meta-analyses of micronutrient intake in community-dwelling older adults showed that thiamine deficiency in food intake in older adults was mainly due to absorption and utilization factors (42). The subgroup analysis based on body mass index indicated that the effect of dietary vitamin B1 intake on heart failure was more significant in overweight people with a BMI of 25–30 kg/m^2^. The possible explanation for this observation is that in individuals with high intake and obesity, glucose consumes most of the serum thiamine levels. Additionally, the surgical treatment of obesity may also impair thiamine absorption. Therefore, it appears that extra thiamine intake is needed for patients with any level of obesity (12). One study showed that smokers suffer from vitamin B1 deficiency more often than nonsmokers. Passive smoking can also affect their eating habits and nutritional status. Therefore, the poor nutritional status of smokers may lead to the appearance or deterioration of various conditions (cardiovascular disease, cancer, cataract, osteoporosis, etc.) related to smoking. Therefore, nutritional monitoring and correction of nutritional deficiencies may be beneficial to the health of smokers and passive smokers, which should be considered in the future (43). In addition, excessive alcohol consumption can affect the cellular transport of vitamin B1 and its intracellular phosphorylation, thereby inhibiting the absorption of vitamins and nutrients. This effect may be crucial for inducing specific nutritional deficiencies (such as thiamine) in alcoholics (13). The results of such prior experiments were consistent with those of our subgroup analysis study. The results of the current study indicated that vitamin B1 was more significantly associated with cardiovascular disease in dyslipidemia subjects. In a cross-sectional study involving diabetic patients, plasma thiamine levels were negatively correlated with TG and LDL levels and positively correlated with HDL levels in patients with DM (44). In healthy older adults, plasma thiamine concentrations were inversely related to total cholesterol concentrations (45).

This study was limited in several aspects. The studies on cardiovascular diseases were based on a cross-sectional design, which does not allow for causal inference. Hence, further prospective studies are needed to confirm these conclusions. Moreover, the number of participants with excessive dietary vitamin B1 intake was small, making it difficult to assess the correlation between excessive intake and cardiovascular diseases. Furthermore, the inclusion criteria for cardiovascular diseases relied on self-reported medical history, and its impact on subtypes and stages of hypertension, coronary heart disease, myocardial infarction, and heart failure was unknown. Therefore, larger clinical studies on different stages and sub-types of cardiovascular diseases are required in the future. Finally, although this analytical model included many covariates, the potential impact of unknown confounders cannot be fully ruled out.

## 5. Conclusion

In this large cohort study, it was observed that higher vitamin B1 intake was associated with a trend toward lower risk of hypertension, heart failure and cardiovascular mortality. In addition, vitamin B1 intake was found to be more protective and pronounced in elderly-aged men, overweight individuals, smokers, drinkers and patients with dyslipidemia. Although the observed correlations require further validation in other populations and intervention studies targeting CVD risk factors, this study highlights the role of vitamin B1 in mitigating cardiovascular diseases. We aim to draw the attention of health policymakers and public health practitioners to include vitamin B1 in an overall healthy basal diet to promote the prevention of cardiovascular diseases.

## Data availability statement

A full list of data sets supporting the results in this research article can be found at: https://wwwn.cdc.gov/nchs/nhanes/.

## Ethics statement

The NCHS Ethics Review Board approved all NHANES programs, and study members or their agents provided informed consent prior to participation (22). Detailed information about NHANES can be found at www.cdc.gov/nchs/nhanes/.

## Author contributions

HW proposed the strategy, implemented the extraction, collation of the data, and drafted the paper. XN and NS validated and analyzed the data. RZ and QW produced the figures. LM, YL, and WZ revised and finalized the manuscript. All authors contributed to the article and approved the submitted version.

## Funding

This work was supported by the grants from the National Natural Science Foundation of China (nos. 81770369 and 82070385).

## Conflict of interest

The authors declare that the research was conducted in the absence of any commercial or financial relationships that could be construed as a potential conflict of interest.

## Publisher’s note

All claims expressed in this article are solely those of the authors and do not necessarily represent those of their affiliated organizations, or those of the publisher, the editors and the reviewers. Any product that may be evaluated in this article, or claim that may be made by its manufacturer, is not guaranteed or endorsed by the publisher.

## Footnotes

^1^ http://www.empowerstats.com
