# Supplementary material for: Association of vitamin B1 with cardiovascular diseases, all-cause and cardiovascular mortality in US adults
Source: Front Nutr. 2023 Aug 31;10:1175961. doi: 10.3389/fnut.2023.1175961 (PMC10502219; doi:10.3389/fnut.2023.1175961)
Supplement: Supplementary file 1 [file Table_1.DOC]

**Table S1 Association between vitamin B1 intake and cardiovascular diseases, all-cause mortality and cardiovascular mortality as categorized by age**

| **Subgroup** | **N** | **HTN** | **CHD** | **MI** | **HF** | **ACM** | **CVDM** |
| --- | --- | --- | --- | --- | --- | --- | --- |
| **Age(years old)** |  |  |  |  |  |  |  |
| **<50** | 13821 | 0.96 (0.91, 1.01) 0.100 | 1.05 (0.75, 1.46) 0.778 | 0.97 (0.74, 1.28) 0.836 | 1.19 (0.91, 1.55) 0.215 | 0.90 (0.79, 1.04) 0.161 | 0.80 (0.58, 1.12) 0.197 |
| **≥50** | 14137 | **0.94 (0.91, 0.98) 0.002** | 1.01 (0.91, 1.12) 0.854 | 1.00 (0.90, 1.11) 0.958 | **0.79 (0.69, 0.91) 0.001** | 1.06 (1.00, 1.12) 0.040 | 0.95 (0.84, 1.07) 0.404 |
| **<50** |  |  |  |  |  |  |  |
| Q1 | 3185 | 1.0 | 1.0 | 1.0 | 1.0 | 1.0 | 1.0 |
| Q2 | 3213 | 1.04 (0.93, 1.16) 0.513 | 0.62 (0.30, 1.26) 0.187 | 0.67 (0.39, 1.16) 0.151 | 1.05 (0.57, 1.95) 0.878 | 0.69 (0.51, 0.93) 0.014 | **0.49 (0.25, 0.96) 0.038** |
| Q3 | 3444 | 1.05 (0.94, 1.18) 0.357 | 0.93 (0.48, 1.83) 0.845 | 0.72 (0.41, 1.26) 0.254 | 1.27 (0.69, 2.36) 0.444 | 0.83 (0.62, 1.11) 0.214 | 0.61 (0.32, 1.17) 0.138 |
| Q4 | 3979 | 0.94 (0.83, 1.07) 0.339 | 0.85 (0.40, 1.82) 0.674 | 0.71 (0.38, 1.32) 0.283 | 1.57 (0.80, 3.08) 0.193 | 0.63 (0.45, 0.87) 0.005 | 0.55 (0.27, 1.10) 0.089 |
| **≥50** |  |  |  |  |  |  |  |
| Q1 | 3794 | 1.0 | 1.0 | 1.0 | 1.0 | 1.0 | 1.0 |
| Q2 | 3781 | 1.01 (0.95, 1.07) 0.806 | **1.24 (1.05, 1.47) 0.013** | 1.06 (0.90, 1.26) 0.465 | 1.06 (0.88, 1.28) 0.517 | 1.02 (0.93, 1.12) 0.604 | 1.07 (0.90, 1.29) 0.439 |
| Q3 | 3545 | 1.03 (0.97, 1.10) 0.344 | **1.24 (1.03, 1.49) 0.022** | 1.03 (0.86, 1.24) 0.734 | 0.87 (0.70, 1.08) 0.209 | **1.13 (1.02, 1.25) 0.022** | 1.13 (0.93, 1.38) 0.225 |
| Q4 | 3017 | 0.93 (0.86, 1.00) 0.054 | 1.19 (0.97, 1.47) 0.103 | 0.96 (0.77, 1.18) 0.676 | **0.73 (0.56, 0.95) 0.019** | 1.12 (1.00, 1.27) 0.055 | 0.93 (0.73, 1.19) 0.568 |

Multivariable model is adjusted for sex, level of education, BMI, smoking history, drinking history, aspirin use, diabetes mellitus, poverty to income ratio, physical activity, Total energy intake, TC, TG, HDL
