# Supplementary material for: Association of vitamin B1 with cardiovascular diseases, all-cause and cardiovascular mortality in US adults
Source: Front Nutr. 2023 Aug 31;10:1175961. doi: 10.3389/fnut.2023.1175961 (PMC10502219; doi:10.3389/fnut.2023.1175961)
Supplement: Supplementary file 2 [file Table_2.DOC]

**Table S2 Association between vitamin B1 intake and cardiovascular diseases, all-cause mortality and cardiovascular mortality as categorized by sex**

| **Subgroup** | **N** | **HTN** | **CHD** | **MI** | **HF** | **ACM** | **CVDM** |
| --- | --- | --- | --- | --- | --- | --- | --- |
| **Sex** |  |  |  |  |  |  |  |
| **Male** | 13840 | **0.91 (0.88, 0.95) <0.001** | 0.97 (0.88, 1.09) 0.644 | 0.95 (0.85, 1.07) 0.410 | **0.80 (0.68, 0.94) 0.005** | 0.94 (0.88, 1.00) 0.062 | **0.83 (0.72, 0.96) 0.010** |
| **Female** | 14118 | **0.94 (0.90, 0.99) 0.023** | 0.89 (0.72, 1.11) 0.303 | 0.91 (0.73, 1.14) 0.409 | 0.81 (0.64, 1.02) 0.079 | 0.99 (0.90, 1.09) 0.817 | 0.86 (0.69, 1.08) 0.190 |
| **Male** |  |  |  |  |  |  |  |
| Q1 | 2254 | 1.0 | 1.0 | 1.0 | 1.0 | 1.0 | 1.0 |
| Q2 | 2902 | 1.00 (0.92, 1.08) 0.970 | 1.23 (0.99, 1.53) 0.057 | 1.08 (0.87, 1.33) 0.487 | 1.02 (0.79, 1.31) 0.875 | 0.96 (0.84, 1.09) 0.502 | 1.05 (0.83, 1.34) 0.668 |
| Q3 | 3622 | 0.95 (0.87, 1.03) 0.196 | 1.14 (0.92, 1.43) 0.236 | 0.96 (0.77, 1.20) 0.741 | 0.89 (0.68, 1.17) 0.414 | 0.92 (0.81, 1.05) 0.233 | 0.87 (0.68, 1.13) 0.300 |
| Q4 | 5062 | **0.88 (0.81, 0.96) 0.005** | 1.12 (0.88, 1.43) 0.357 | 0.88 (0.69, 1.12) 0.302 | **0.73 (0.54, 1.00) 0.047** | 0.86 (0.75, 1.00) 0.048 | 0.76 (0.57, 1.01) 0.058 |
| **Female** |  |  |  |  |  |  |  |
| Q1 | 4725 | 1.0 | 1.0 | 1.0 | 1.0 | 1.0 | 1.0 |
| Q2 | 4092 | **0.92 (0.86, 0.99) 0.019** | 0.96 (0.74, 1.24) 0.761 | 0.85 (0.66, 1.10) 0.220 | 0.94 (0.73, 1.21) 0.636 | 0.80 (0.70, 0.90) **<0.001** | **0.73 (0.56, 0.94) 0.017** |
| Q3 | 3367 | 1.00 (0.93, 1.08) 0.926 | 1.05 (0.77, 1.42) 0.775 | 0.93 (0.69, 1.27) 0.657 | **0.72 (0.52, 1.00) 0.050** | 0.92 (0.80, 1.07) 0.272 | 0.99 (0.74, 1.34) 0.972 |
| Q4 | 1934 | **0.87 (0.79, 0.96) 0.004** | 1.00 (0.68, 1.48) 0.992 | 0.92 (0.61, 1.37) 0.672 | 0.85 (0.57, 1.28) 0.446 | 0.93 (0.78, 1.12) 0.464 | 0.71 (0.47, 1.07) 0.104 |

Multivariable model is adjusted for age, level of education, BMI, smoking history, drinking history, aspirin use, diabetes mellitus, poverty to income ratio, physical activity, Total energy intake, TC, TG, HDL
