# Supplementary material for: Association of vitamin B1 with cardiovascular diseases, all-cause and cardiovascular mortality in US adults
Source: Front Nutr. 2023 Aug 31;10:1175961. doi: 10.3389/fnut.2023.1175961 (PMC10502219; doi:10.3389/fnut.2023.1175961)
Supplement: Supplementary file 3 [file Table_3.DOC]

### **Table S3 Association between vitamin B1 intake and cardiovascular diseases, all-cause mortality and cardiovascular mortality as categorized by BMI**

| **Subgroup** | **N** | **HTN** | **CHD** | **MI** | **HF** | **ACM** | **CVDM** |
| --- | --- | --- | --- | --- | --- | --- | --- |
| **BMI(kg/m^2^)** |  |  |  |  |  |  |  |
| **<25** | 7947 | **0.89 (0.83, 0.95) 0.001** | 0.92 (0.74, 1.12) 0.400 | 1.04 (0.85, 1.27) 0.711 | 1.00 (0.76, 1.31) 0.981 | 0.91 (0.83, 1.01) 0.074 | **0.75 (0.59, 0.95) 0.017** |
| **25-30** | 9434 | **0.91 (0.86, 0.96) <0.001** | 0.87 (0.74, 1.03) 0.109 | **0.82 (0.68, 0.98) 0.027** | **0.58 (0.44, 0.75) <0.001** | 0.95 (0.86, 1.03) 0.222 | 0.84 (0.69, 1.01) 0.059 |
| **≥30** | 10577 | **0.95 (0.91, 1.00) 0.039** | 1.07 (0.92, 1.24) 0.392 | 1.03 (0.89, 1.20) 0.666 | 0.89 (0.74, 1.06) 0.194 | 1.01 (0.91, 1.11) 0.922 | 0.89 (0.72, 1.09) 0.268 |
| **<25** |  |  |  |  |  |  |  |
| Q1 | 1862 | 1.0 | 1.0 | 1.0 | 1.0 | 1.0 | 1.0 |
| Q2 | 1902 | 0.93 (0.83, 1.05) 0.245 | 1.01 (0.70, 1.47) 0.950 | 0.74 (0.51, 1.07) 0.107 | 0.74 (0.48, 1.15) 0.186 | 0.89 (0.75, 1.05) 0.151 | 0.95 (0.67, 1.34) 0.762 |
| Q3 | 1999 | 0.92 (0.81, 1.04) 0.187 | 1.02 (0.70, 1.50) 0.904 | 0.89 (0.62, 1.28) 0.528 | 0.81 (0.51, 1.28) 0.363 | 0.86 (0.72, 1.02) 0.075 | **0.64 (0.43, 0.94) 0.022** |
| Q4 | 2184 | 0.83 (0.72, 0.96) 0.011 | 0.90 (0.58, 1.40) 0.633 | 0.83 (0.54, 1.27) 0.386 | 0.87 (0.52, 1.48) 0.613 | 0.83 (0.68, 1.01) 0.060 | **0.62 (0.40, 0.97) 0.035** |
| **25-30** |  |  |  |  |  |  |  |
| Q1 | 2276 | 1.0 | 1.0 | 1.0 | 1.0 | 1.0 | 1.0 |
| Q2 | 2368 | 0.93 (0.85, 1.01) 0.095 | 1.19 (0.90, 1.57) 0.217 | 1.14 (0.86, 1.51) 0.356 | 1.14 (0.82, 1.57) 0.4302 | 0.87 (0.75, 1.01) 0.059 | 0.93 (0.70, 1.24) 0.616 |
| Q3 | 2360 | 0.95 (0.87, 1.05) 0.330 | 1.01 (0.75, 1.37) 0.942 | 0.98 (0.72, 1.33) 0.891 | 0.80 (0.55, 1.17) 0.2478 | 0.92 (0.78, 1.08) 0.305 | 0.97 (0.71, 1.32) 0.834 |
| Q4 | 2430 | **0.82 (0.73, 0.92) 0.001** | 0.98 (0.69, 1.38) 0.892 | **0.64 (0.44, 0.94) 0.022** | **0.49 (0.30, 0.80) 0.004** | **0.79 (0.65, 0.95) 0.014** | 0.76 (0.52, 1.10) 0.148 |
| **≥30** |  |  |  |  |  |  |  |
| Q1 | 2841 | 1.0 | 1.0 | 1.0 | 1.0 | 1.0 | 1.0 |
| Q2 | 2724 | 1.01 (0.94, 1.09) 0.728 | 1.08 (0.84, 1.38) 0.542 | 0.93 (0.74, 1.17) 0.516 | 1.00 (0.78, 1.28) 0.998 | 0.89 (0.77, 1.04) 0.142 | 0.82 (0.62, 1.10) 0.186 |
| Q3 | 2630 | 1.01 (0.93, 1.09) 0.809 | 1.17 (0.90, 1.53) 0.251 | 0.85 (0.65, 1.10) 0.216 | 0.85 (0.64, 1.14) 0.280 | 0.96 (0.81, 1.13) 0.588 | 0.96 (0.70, 1.31) 0.795 |
| Q4 | 2382 | 0.94 (0.86, 1.03) 0.195 | 1.23 (0.91, 1.67) 0.179 | 1.05 (0.79, 1.40) 0.729 | 0.89 (0.64, 1.24) 0.478 | 1.02 (0.84, 1.23) 0.873 | 0.75 (0.50, 1.10) 0.142 |

Multivariable model is adjusted for age, sex, level of education, smoking history, drinking history, aspirin use, diabetes mellitus, poverty to income ratio, physical activity, Total energy intake, TC, TG, HDL
