# Supplementary material for: Association of vitamin B1 with cardiovascular diseases, all-cause and cardiovascular mortality in US adults
Source: Front Nutr. 2023 Aug 31;10:1175961. doi: 10.3389/fnut.2023.1175961 (PMC10502219; doi:10.3389/fnut.2023.1175961)
Supplement: Supplementary file 4 [file Table_4.DOC]

### **Table S4 Association between vitamin B1 intake and cardiovascular diseases, all-cause mortality and cardiovascular mortality as categorized by smoking history**

| **Subgroup** | **N** | **HTN** | **CHD** | **MI** | **HF** | **ACM** | **CVDM** |
| --- | --- | --- | --- | --- | --- | --- | --- |
| **Smoking history** |  |  |  |  |  |  |  |
| No | 15037 | **0.92 (0.88, 0.97) <0.001** | 0.90 (0.76, 1.07) 0.248 | 1.07 (0.90, 1.27) 0.430 | **0.74 (0.58, 0.93) 0.011** | 1.00 (0.92, 1.10) 0.933 | 0.99 (0.83, 1.19) 0.915 |
| **Yes** | 12921 | **0.93 (0.89, 0.97) <0.001** | 1.00 (0.89, 1.12) 0.987 | 0.91 (0.81, 1.03) 0.148 | **0.85 (0.73, 0.99) 0.043** | 0.94 (0.88, 1.01) 0.077 | **0.76 (0.65, 0.89) <0.001** |
| **No** |  |  |  |  |  |  |  |
| Q1 | 3683 | 1.0 | 1.0 | 1.0 | 1.0 | 1.0 | 1.0 |
| Q2 | 3817 | 1.01 (0.94, 1.08) 0.877 | 1.20 (0.92, 1.57) 0.181 | 1.10 (0.83, 1.45) 0.514 | 0.91 (0.68, 1.20) 0.493 | **0.85 (0.74, 0.99) 0.032** | 0.83 (0.63, 1.10) 0.192 |
| Q3 | 3875 | 0.98 (0.90, 1.06) 0.558 | 1.05 (0.78, 1.42) 0.736 | 1.04 (0.76, 1.41) 0.825 | 0.83 (0.60, 1.15) 0.268 | 1.02 (0.87, 1.18) 0.832 | 1.06 (0.79, 1.43) 0.687 |
| Q4 | 3662 | **0.89 (0.81, 0.97) 0.011** | 0.88 (0.62, 1.26) 0.498 | 1.07 (0.75, 1.52) 0.727 | **0.57 (0.37, 0.88) 0.011** | 0.92 (0.76, 1.11) 0.384 | 1.00 (0.70, 1.44) 0.982 |
| **Yes** |  |  |  |  |  |  |  |
| Q1 | 3296 | 1.0 | 1.0 | 1.0 | 1.0 | 1.0 | 1.0 |
| Q2 | 3177 | 0.93 (0.87, 1.00) 0.055 | 1.05 (0.85, 1.29) 0.645 | 0.89 (0.73, 1.08) 0.231 | 1.08 (0.86, 1.36) 0.507 | 0.91 (0.81, 1.02) 0.090 | 0.92 (0.74, 1.16) 0.492 |
| Q3 | 3114 | 0.99 (0.92, 1.07) 0.828 | 1.11 (0.89, 1.39) 0.337 | 0.87 (0.70, 1.07) 0.190 | 0.87 (0.67, 1.12) 0.281 | **0.88 (0.78, 1.00) 0.042** | 0.79 (0.62, 1.01) 0.065 |
| Q4 | 3334 | **0.89 (0.81, 0.97) 0.006** | 1.18 (0.92, 1.51) 0.193 | 0.78 (0.61, 1.00) 0.052 | **0.89 (0.66, 1.20) 0.440** | **0.87 (0.75, 1.00) 0.049** | **0.59 (0.44, 0.80) <0.001** |

Multivariable model is adjusted for age, sex, level of education, BMI, drinking history, aspirin use, diabetes mellitus, poverty to income ratio, physical activity, Total energy intake, TC, TG, HDL
