# Supplementary material for: Association of vitamin B1 with cardiovascular diseases, all-cause and cardiovascular mortality in US adults
Source: Front Nutr. 2023 Aug 31;10:1175961. doi: 10.3389/fnut.2023.1175961 (PMC10502219; doi:10.3389/fnut.2023.1175961)
Supplement: Supplementary file 5 [file Table_5.DOC]

### **Table S5 Association between vitamin B1 intake and cardiovascular diseases, all-cause mortality and cardiovascular mortality as categorized by drinking history**

| **Subgroup** | **N** | **HTN** | **CHD** | **MI** | **HF** | **ACM** | **CVDM** |
| --- | --- | --- | --- | --- | --- | --- | --- |
| **Drinking history** |  |  |  |  |  |  |  |
| No | 8599 | **0.93 (0.88, 0.98) 0.010** | 0.96 (0.81, 1.15) 0.676 | 0.99 (0.83, 1.19) 0.947 | **0.76 (0.60, 0.95) 0.018** | 0.94 (0.85, 1.04) 0.251 | 0.90 (0.74, 1.10) 0.313 |
| **Yes** | 19359 | **0.92 (0.89, 0.96) <0.001** | 0.97 (0.87, 1.10) 0.666 | 0.94 (0.83, 1.06) 0.301 | **0.84 (0.72, 0.98) 0.029** | 0.96 (0.90, 1.03) 0.260 | **0.81 (0.70, 0.94) 0.005** |
| **No** |  |  |  |  |  |  |  |
| Q1 | 2574 | 1.0 | 1.0 | 1.0 | 1.0 | 1.0 | 1.0 |
| Q2 | 2259 | 0.99 (0.91, 1.07) 0.776 | 1.01 (0.77, 1.33) 0.942 | 0.92 (0.70, 1.22) 0.556 | 0.88 (0.67, 1.17) 0.382 | 0.81 (0.69, 0.94) 0.005 | 0.79 (0.59, 1.05) 0.105 |
| Q3 | 2059 | 1.00 (0.92, 1.10) 0.927 | 1.14 (0.86, 1.53) 0.365 | 1.05 (0.78, 1.41) 0.769 | 0.77 (0.55, 1.06) 0.112 | 0.95 (0.81, 1.12) 0.531 | 0.88 (0.64, 1.20) 0.418 |
| Q4 | 1707 | **0.89 (0.80, 1.00) 0.050** | 0.99 (0.69, 1.41) 0.951 | 0.91 (0.63, 1.32) 0.632 | 0.67 (0.44, 1.00) 0.052 | 0.90 (0.73, 1.09) 0.285 | 0.78 (0.53, 1.15) 0.213 |
| **Yes** |  |  |  |  |  |  |  |
| Q1 | 4405 | 1.0 | 1.0 | 1.0 | 1.0 | 1.0 | 1.0 |
| Q2 | 4735 | 0.95 (0.89, 1.01) 0.096 | 1.13 (0.92, 1.39) 0.241 | 0.96 (0.79, 1.16) 0.654 | 1.07 (0.85, 1.34) 0.580 | 0.92 (0.82, 1.02) 0.126 | 0.93 (0.75, 1.16) 0.543 |
| Q3 | 4930 | 0.96 (0.90, 1.03) 0.302 | 1.04 (0.83, 1.30) 0.739 | 0.84 (0.68, 1.04) 0.119 | 0.87 (0.67, 1.13) 0.307 | 0.91 (0.81, 1.02) 0.110 | 0.88 (0.70, 1.12) 0.31 |
| Q4 | 5289 | **0.88 (0.81, 0.95) 0.001** | 1.11 (0.87, 1.42) 0.415 | 0.83 (0.65, 1.06) 0.140 | 0.82 (0.61, 1.12) 0.211 | 0.88 (0.76, 1.00) 0.055 | **0.70 (0.53, 0.93) 0.015** |

Multivariable model is adjusted for age, sex, level of education, BMI, smoking history, aspirin use, diabetes mellitus, poverty to income ratio, physical activity, Total energy intake, TC, TG, HDL
