# Supplementary material for: Association of vitamin B1 with cardiovascular diseases, all-cause and cardiovascular mortality in US adults
Source: Front Nutr. 2023 Aug 31;10:1175961. doi: 10.3389/fnut.2023.1175961 (PMC10502219; doi:10.3389/fnut.2023.1175961)
Supplement: Supplementary file 6 [file Table_6.DOC]

**Table S6 Association between vitamin B1 intake and cardiovascular diseases, all-cause mortality and cardiovascular mortality as categorized by blood lipid**

| **Subgroup** | **N** | **HTN** | **CHD** | **MI** | **HF** | **ACM** | **CVDM** |
| --- | --- | --- | --- | --- | --- | --- | --- |
| **Blood lipid** |  |  |  |  |  |  |  |
| [**Ortholiposis**](javascript:;) | 11338 | **0.92 (0.87, 0.97) 0.001** | 0.95 (0.81, 1.10) 0.490 | 0.97 (0.83, 1.13) 0.677 | **0.81 (0.66, 1.00) 0.048** | 0.95 (0.87, 1.04) 0.294 | 0.90 (0.74, 1.10) 0.308 |
| [**Dyslipidemia**](javascript:;) | 16620 | **0.92 (0.89, 0.96) <0.001** | 1.02 (0.90, 1.16) 0.711 | 0.99 (0.87, 1.12) 0.818 | **0.84 (0.71, 0.99) 0.036** | 0.95 (0.89, 1.02) 0.168 | **0.81 (0.70, 0.94) 0.005** |
| [**Ortholiposis**](javascript:;) |  |  |  |  |  |  |  |
| Q1 | 2799 | 1.0 | 1.0 | 1.0 | 1.0 | 1.0 | 1.0 |
| Q2 | 2816 | 0.98 (0.90, 1.07) 0.612 | 1.12 (0.86, 1.46) 0.405 | 0.93 (0.72, 1.21) 0.608 | 0.84 (0.63, 1.13) 0.251 | 0.87 (0.75, 1.01) 0.065 | 0.94 (0.69, 1.26) 0.667 |
| Q3 | 2848 | 0.96 (0.87, 1.05) 0.357 | 1.16 (0.88, 1.53) 0.297 | 1.08 (0.82, 1.42) 0.585 | 0.80 (0.58, 1.11) 0.188 | **0.84 (0.71, 0.98) 0.030** | 0.84 (0.60, 1.17) 0.302 |
| Q4 | 2875 | **0.89 (0.80, 0.99) 0.030** | 1.03 (0.75, 1.42) 0.861 | 0.91 (0.66, 1.25) 0.556 | 0.73 (0.50, 1.08) 0.112 | **0.82 (0.68, 0.99) 0.035** | 0.87 (0.60, 1.27) 0.479 |
| [**Dyslipidemia**](javascript:;) |  |  |  |  |  |  |  |
| Q1 | 4180 | 1.0 | 1.0 | 1.0 | 1.0 | 1.0 | 1.0 |
| Q2 | 4178 | 0.97 (0.92, 1.04) 0.417 | 1.13 (0.92, 1.40) 0.247 | 1.00 (0.82, 1.23) 0.974 | 1.14 (0.91, 1.43) 0.243 | **0.89 (0.79, 0.99) 0.030** | 0.86 (0.69, 1.07) 0.172 |
| Q3 | 4141 | 1.00 (0.94, 1.07) 0.986 | 1.06 (0.84, 1.34) 0.610 | 0.83 (0.66, 1.05) 0.121 | 0.90 (0.69, 1.17) 0.419 | 0.97 (0.86, 1.09) 0.561 | 0.91 (0.72, 1.15) 0.426 |
| Q4 | 4121 | **0.88 (0.81, 0.95) 0.002** | 1.23 (0.95, 1.60) 0.121 | 0.90 (0.69, 1.17) 0.440 | 0.83 (0.60, 1.14) 0.244 | 0.90 (0.79, 1.04) 0.158 | **0.65 (0.48, 0.87) 0.003** |

Multivariable model is adjusted for age, sex, level of education, BMI, smoking history, drinking history, aspirin use, diabetes mellitus, poverty to income ratio, physical activity, Total energy intake
