# Supplementary material for: Association of vitamin B1 with cardiovascular diseases, all-cause and cardiovascular mortality in US adults
Source: Front Nutr. 2023 Aug 31;10:1175961. doi: 10.3389/fnut.2023.1175961 (PMC10502219; doi:10.3389/fnut.2023.1175961)
Supplement: Supplementary file 7 [file Table_7.DOC]

**Table S7a Association between vitamin B1 intake and cardiovascular diseases by excluding deaths during 1-year follow-up baseline**

|  | **Model 1**  **OR（95% CI） p value** | **Model 2**  **OR（95% CI）p value** | **Model 3**  **OR（95% CI） p value** |
| --- | --- | --- | --- |
| **HTN** |  |  |  |
| VitaminB1(mg/d) | **0.83 (0.80, 0.85) <0.001** | **0.95 (0.91, 0.98) 0.003** | **0.95 (0.90, 0.99) 0.016** |
| VitaminB1(mg/d) quartiles |  |  |  |
| Q1 | 1.0 | 1.0 | 1.0 |
| Q2 | **0.88 (0.82, 0.94) <0.001** | **0.84 (0.78, 0.91) <0.001** | **0.82 (0.76, 0.89) <0.001** |
| Q3 | **0.82 (0.77, 0.88) <0.001** | **0.86 (0.80, 0.93) <0.001** | **0.83 (0.77, 0.91) <0.001** |
| Q4 | **0.68 (0.63, 0.72) <0.001** | **0.85 (0.78, 0.92) <0.001** | **0.84 (0.76, 0.92) <0.001** |
| **CHD** |  |  |  |
| VitaminB1(mg/d) | **0.91 (0.85, 0.99) 0.021** | 0.93 (0.85, 1.02) 0.117 | 1.02 (0.92, 1.13) 0.742 |
| VitaminB1(mg/d) quartiles |  |  |  |
| Q1 | 1.0 | 1.0 | 1.0 |
| Q2 | 1.07 (0.91, 1.27) 0.393 | 0.99 (0.83, 1.17) 0.881 | 1.02 (0.85, 1.22) 0.831 |
| Q3 | 1.05 (0.89, 1.24) 0.5823 | 0.94 (0.79, 1.12) 0.520 | 1.05 (0.86, 1.27) 0.644 |
| Q4 | 0.92 (0.78, 1.09) 0.350 | 0.92 (0.77, 1.11) 0.379 | 1.11 (0.89, 1.38) 0.363 |
| **MI** |  |  |  |
| VitaminB1(mg/d) | **0.86 (0.80, 0.93) <0.001** | **0.88 (0.80, 0.96) 0.004** | 0.99 (0.89, 1.10) 0.877 |
| VitaminB1(mg/d) quartiles |  |  |  |
| Q1 | 1.0 | 1.0 | 1.0 |
| Q2 | 0.93 (0.80, 1.10) 0.404 | 0.87 (0.73, 1.02) 0.089 | 0.91 (0.76, 1.08) 0.273 |
| Q3 | **0.85 (0.72, 1.00) 0.047** | **0.77 (0.65, 0.92) 0.003** | 0.87 (0.72, 1.05) 0.151 |
| Q4 | **0.72 (0.61, 0.85) <0.001** | **0.71 (0.59, 0.85) <0.001** | 0.87 (0.70, 1.08) 0.219 |
| **HF** |  |  |  |
| VitaminB1(mg/d) | **0.71 (0.64, 0.78) <0.001** | **0.77 (0.69, 0.86) <0.001** | **0.84 (0.73, 0.97) 0.016** |
| VitaminB1(mg/d) quartiles |  |  |  |
| Q1 | 1.0 | 1.0 | 1.0 |
| Q2 | 0.95 (0.80, 1.13) 0.571 | 0.93 (0.78, 1.12) 0.458 | 0.99 (0.81, 1.20) 0.899 |
| Q3 | **0.72 (0.59, 0.87) <0.001** | **0.73 (0.60, 0.89) 0.002** | 0.83 (0.67, 1.03) 0.095 |
| Q4 | **0.55 (0.45, 0.68) <0.001** | **0.65 (0.52, 0.81) <0.001** | 0.80 (0.62, 1.04) 0.093 |

## Model 1: No adjustments made for confounding factors

## Model 2: Adjustments made for age, sex, level of education

Model 3: Adjustments made for age, sex, level of education, BMI, smoking history, drinking history, aspirin use, diabetes mellitus, poverty to income ratio, physical activity, Total energy intake, TC, TG, HDL

**Table S7b Association between vitamin B1 intake and all-cause mortality and cardiovascular mortality by excluding deaths during 1-year follow-up baseline**

|  | **Model 1**  **HR（95% CI） p value** | **Model 2**  **HR（95% CI）p value** | **Model 3**  **HR（95% CI） p value** |
| --- | --- | --- | --- |
| **ACM** |  |  |  |
| VitaminB1(mg/d) | **0.84 (0.80, 0.87) <0.001** | **0.95 (0.91, 1.00) 0.035** | 0.96 (0.90, 1.01) 0.113 |
| VitaminB1(mg/d) quartiles |  |  |  |
| Q1 | 1.0 | 1.0 | 1.0 |
| Q2 | **0.88 (0.81, 0.96) 0.004** | **0.89 (0.81, 0.97) 0.007** | **0.88 (0.81, 0.97) 0.008** |
| Q3 | **0.89 (0.81, 0.97) 0.007** | 0.92 (0.84, 1.01) 0.069 | 0.94 (0.85, 1.03) 0.190 |
| Q4 | **0.68 (0.62, 0.74) <0.001** | **0.86 (0.78, 0.95) 0.003** | **0.88 (0.79, 0.99) 0.032** |
| **CVDM** |  |  |  |
| VitaminB1(mg/d) | **0.77 (0.70, 0.84) <0.001** | **0.85 (0.77, 0.94) 0.001** | **0.83 (0.74, 0.94) 0.003** |
| VitaminB1(mg/d) quartiles |  |  |  |
| Q1 | 1.0 | 1.0 | 1.0 |
| Q2 | 0.90 (0.75, 1.06) 0.209 | 0.89 (0.75, 1.06) 0.180 | 0.87 (0.73, 1.04) 0.135 |
| Q3 | 0.89 (0.74, 1.05) 0.172 | 0.88 (0.74, 1.06) 0.175 | 0.89 (0.73, 1.08) 0.236 |
| Q4 | **0.58 (0.48, 0.70) <0.001** | **0.71 (0.58, 0.87) <0.001** | **0.71 (0.56, 0.90) 0.004** |

## Model 1: No adjustments made for confounding factors

## Model 2: Adjustments made for age, sex, level of education

## Model 3: Adjustments made for age, sex, level of education, BMI, smoking history, drinking history, aspirin use, diabetes mellitus, poverty to income ratio, physical activity, Total energy intake, TC, TG, HDL
