# Supplementary figures and images for: Association of vitamin B1 with cardiovascular diseases, all-cause and cardiovascular mortality in US adults
Source: Front Nutr. 2023 Aug 31;10:1175961. doi: 10.3389/fnut.2023.1175961 (PMC10502219; doi:10.3389/fnut.2023.1175961)

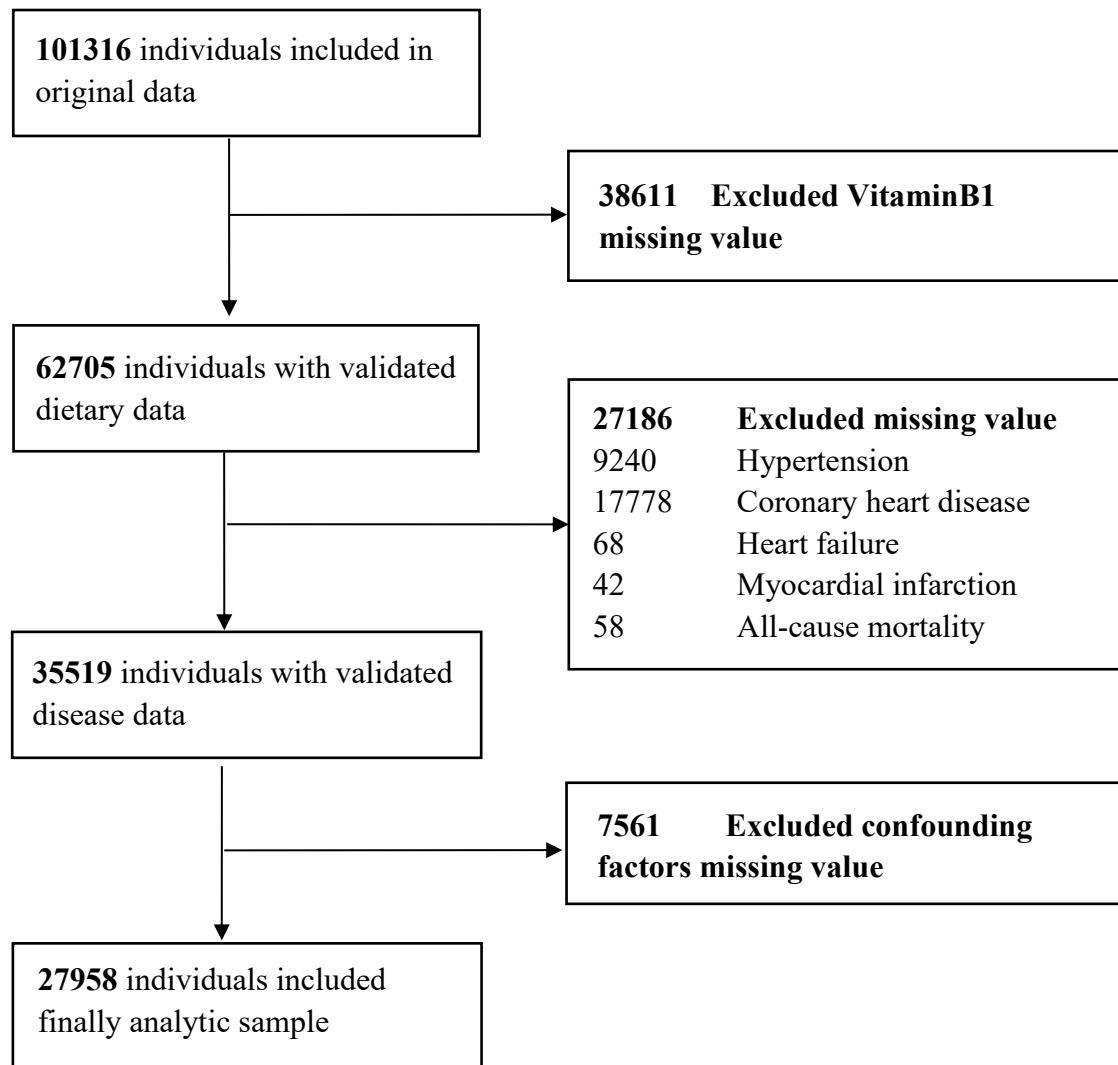

**Figure S1: Participant flow diagram**

Supplement: Supplementary file 8 [file Data_Sheet_1.PDF]
